# Impact of Matrix Metalloproteinase-9 during Periodontitis and Cardiovascular Diseases

**DOI:** 10.3390/molecules26061777

**Published:** 2021-03-22

**Authors:** Gaetano Isola, Alessandro Polizzi, Vincenzo Ronsivalle, Angela Alibrandi, Giuseppe Palazzo, Antonino Lo Giudice

**Affiliations:** 1 Department of General Surgery and Surgical-Medical Specialties, School of Dentistry, University of Catania, 95124 Catania, Italy; alexpoli345@gmail.com (A.P.); vincenzo.ronsivalle@hotmail.it (V.R.); gpalazzo@unict.it (G.P.); nino.logiudice@gmail.com (A.L.G.); 2 Department of Economical, Business and Environmental Sciences and Quantitative Methods, University of Messina, 98122 Messina, Italy; aalibrandi@unime.it

**Keywords:** periodontitis, cardiovascular disease, matrix metalloproteinases, serum, saliva, clinical trial

## Abstract

Matrix metalloproteinase-9 (MMP-9) has been shown to play a key role in endothelial function and perhaps pivotal in the correlation between periodontal disease and cardiovascular disease (CVD). For the study, the impact of MMP-9 of periodontitis and CVD on serum and saliva concentrations was analyzed. For the study patients with periodontitis (n = 31), CVD (n = 31), periodontitis + CVD (n = 31), and healthy patients (n = 31) were enrolled. Clinical and demographic characteristics as well as serum and salivary MMP-9 were evaluated. MMP-9 concentrations in serum and saliva were statistically elevated in patients with CVD (*p* < 0.01) and in patients with periodontitis plus CVD (*p* < 0.001) compared to patients with periodontitis and healthy subjects. Multivariate regression analysis showed that c-reactive protein (hs-CRP) was the only significant predictor for MMP-9 serum (*p* < 0.001), whereas hs-CRP (*p* < 0.001) and total cholesterol (*p* = 0.029) were the statistically significant salivary MMP-9 predictors. This study evidenced that patients with CVD and periodontitis + CVD presented elevated MMP-9 concentrations in serum and saliva compared to patients with periodontitis and healthy subjects. Furthermore, hs-CRP was a negative predictor of serum and salivary MMP-9.

## 1. Introduction

Periodontitis is a multifactorial inflammatory disease that, following the insult of periodontal pathogens, results in the breakdown of the supportive periodontal tissues that can evolve in tooth loss if not adequately treated [1]. There is increasing evidence in the literature linking periodontitis to some systemic diseases, including diabetes, endothelial dysfunction, and cardiovascular disease (CVD) [2,3,4].

During the last decades, several common mediators have been demonstrated to be involved in the pathogenesis of both periodontitis and CVD through the secretion and release of toxic metabolites, [5,6] and proinflammatory cytokines such as interleukins (IL) and matrix metalloproteinases (MMPs) [7,8,9]. 

Periodontitis and CVD have been shown to share common etiopathogenetic mechanisms. More specifically, severe periodontitis is independently and significantly associated with all-cause and CVD mortality in several different populations [10,11]. There is evidence from epidemiological studies that periodontitis patients exhibit significant endothelial dysfunction, measured by flow-mediated dilation (FMD), arterial stiffness (e.g., pulse wave velocity), and a significantly greater carotid intima-media (cIMT) thickness and elevated arterial calcification scores [11]. There was one imaging study that associated high levels of antibodies against periodontal pathogens and a lower extent of positive atheromatous plaque remodeling [12]. Moreover, antigens derived from oral periodontal pathogens in atherothrombotic tissues have been identified in studies on DNA and RNA [13]. Studies have attempted to correlate the presence of oral bacteria in atherothrombotic tissues with subgingival plaque or serum in the same patients and suggested that in periodontitis patients, there is a higher probability of developing CVD or endothelial dysfunction [13,14]. Proposed mechanisms include bacteremia and the associated systemic inflammatory sequelae, including elevations in C-reactive protein and oxidative stress [14].

Furthermore, it has been shown that during periodontitis and CVD there is a significant increase, on both an oral and a systemic level, of various inflammatory mediators, including c-reactive protein (CRP), prostaglandins, IL-1, IL-6, IL-10, and some MMPs following the immune response due to the presence of specific periodontal pathogenic bacteria [13,14,15]. More specifically, several reports have shown that the levels of MMPs are more upregulated during periodontitis compared to gingivitis or oral health, demonstrating that unbalanced MMPs may favor rapid periodontal tissue destruction [16].

Among MMP isoforms, metalloproteinase-9 (MMP-9) has been validated in various pre-clinical models as one of the most frequent mediators present in tissues during the active stages of inflammation progression in patients with either periodontitis [17] or CVD [18]. MMP-9 has been shown to be released by macrophages at the vascular level following exposure to pathogenic bacteria or during host response and has been validated as a subclinical marker of vascular homeostasis [17]. It has been found that MMP-9, during the initial phases of inflammation, regulates some mediators, including IL-1, -6, and -8 and prostaglandins [19,20]. 

Preliminary evidence has demonstrated that the expression of MMP-9 is associated with periodontal tissue damage during active stages of periodontitis [21,22]. In this regard, some reports have demonstrated upregulated MMP-9 in gingival crevicular fluid (GCF) during the initial phase of periodontitis [23,24], with a key role played by MMP-9 in neoangiogenesis associated with the host response to periodontal pathogens [25,26]. Moreover, during periodontitis, it has been hypothesized that MMP-9, together with CRP, may negatively upregulate nitric oxide (NO) [27,28], which in turn may adversely affect the endothelium and arterial vascular tone and finally lead to endothelial dysfunction and augmented CVD risk [29].

For these reasons, there is growing scientific interest in the search for some early markers that may help in assessing the early subclinical risk of periodontitis and CVD.

Therefore, based on this evidence, this study aimed to evaluate the serum and salivary levels of MMP-9 in patients with periodontitis and with CVD, and to investigate the effect of periodontitis and CVD on serum and salivary levels of MMP-9. Furthermore, it was investigated whether possible confounders could mediate associations between MMP-9, periodontitis, and CVD. The null hypothesis to invalidate was that there was no difference in MMP-9 expression and association among the analyzed patients.

## 2. Results

The sample demographic characteristics are represented in Table 1. All patients were Caucasians and were well matched for demographic characteristics.

In comparison to controls subjects, patients with CVD, periodontitis, and periodontitis + CVD had elevated values of hs-CRP (*p* < 0.001). In addition, CVD and periodontitis + CVD patients did not show significant differences in previous CVD episodes. There were no patients with hyperlipidemia in all analyzed groups.

Periodontal characteristics are represented in Table 2. Compared to patients with CVD and control patients, periodontitis and periodontitis + CVD patients exhibited elevated clinical attachment level (CAL), probing depth (PD), bleeding on probing (BOP), and plaque index (PI) and a reduced number of teeth (*p* < 0.001).

**Figure 1:**
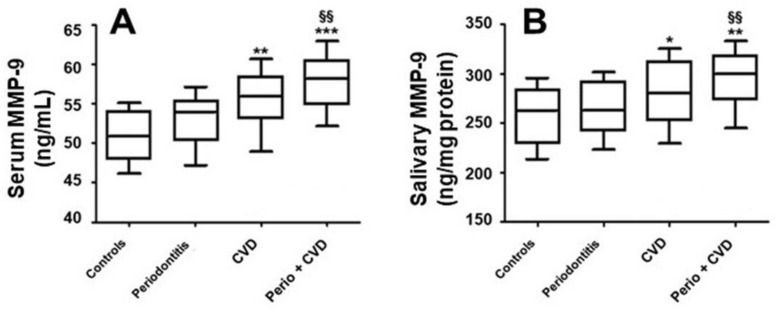
Metalloproteinases-9 (MMP-9) in saliva (**B**) and serum (**A**) (median, 25%; 75% percentiles). §§ *p* < 0.01 differences vs. periodontitis patients. * *p* < 0.05, ** *p* < 0.01, and *** *p* < 0.001 differences vs. healthy controls.

Salivary and serum MMP-9 levels in the analyzed sample are represented in Figure 1. In comparison to healthy controls, MMP-9 concentrations in serum and saliva were statistically elevated in patients with CVD [serum: 51.3 (38.2–64.4) ng/mL; saliva 256.4 (217.4–549.5) ng/mL, *p* < 0.01] and in patients with periodontitis + CVD [serum: 53.8 (38.5–64.7) ng/mL; saliva 265.6 (165.5–586.7) ng/mL, *p* < 0.001]. Furthermore, there was no statistical association between serum and salivary MMP-9 levels (*p* = 0.542).

**Figure 2:**
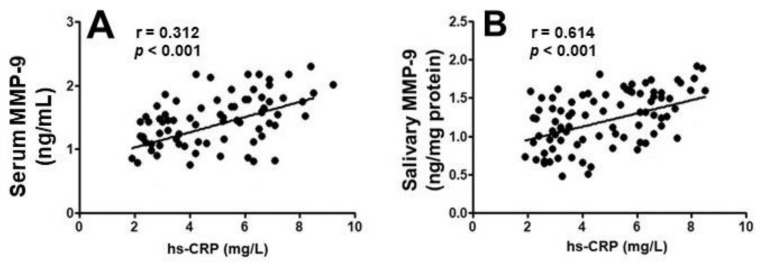
Correlation among MMP-9 concentrations in serum (**A**) and saliva (**B**) with hs-CRP.

The *p* for trend test evidenced that MMP-9 in both serum and saliva were gradually increased in healthy patients and in patients with periodontitis, CVD, and periodontitis + CVD (*p*-trend < 0.001). No statistical association was found in serum and salivary MMP-9 concentrations (rs = 0.199, *p* = 0.112). Furthermore, there was a significant correlation among MMP-9 concentrations in serum and saliva with hs-CRP (rs = 0.312, *p* < 0.001)/(rs = 0.614, *p* < 0.001) (Figure 2).

The univariate regression models applied demonstrated that there was a statistical association among MMP-9 in serum (*p* < 0.001) and saliva (*p* < 0.001), and hs-CRP. 

The subsequent adjusted multivariate linear regression analysis demonstrated that hs-CRP was the statistical predictor for serum MMP-9 concentrations (*p* < 0.001). For salivary MMP-9, total cholesterol (*p* = 0.029) and hs-CRP (*p* < 0.001) were the statistically significant predictors (Table 3).

## 3. Discussion

This study aimed to evaluate the association and effect of periodontitis and CVD on serum and salivary MMP-9 levels. This study found that CVD and periodontitis + CVD presented elevated MMP-9 levels in comparison to periodontitis patients and healthy subjects.

In accordance with what is reported in the present study, other studies on the subject showed high serum levels of MMP-9; furthermore, the presence of such elevated serum levels of MMP-9 has been shown to result in a high and independent risk of increased risk of CVD and myocardial infarction [30,31]. In fact, it was shown that patients with atherosclerosis who had high serum levels of MMP-9 had a high risk of developing CVD and carotid endothelial dysfunction, underlining the essential role played by MMP-9 in the inhibition of NO at the endothelial level [32,33]. Furthermore, evidence in these areas has shown that the possible presence of periodontitis in patients with CVD can represent a strong stimulus to endothelial dysfunction in patients with CVD [34,35]. In this regard, recent research has shown that patients with periodontitis had significantly high serum levels of MMP-9 [12,19] and that good maintenance therapy resulted in a significant reduction in serum levels of MMP-9 in patients with coronary artery disease [36].

Several other pieces of evidence in recent years have also shown that the high increase in MMP-9 during inflammatory diseases was also directly due (cause/effect) to an increase in serum levels of hs-CRP [36,37,38]. According to what was reported in the present study, patients with CVD and periodontitis + CVD had high levels of MMP-9, associating the hypothesis that conditions that favor an increase in NO and oxidative stress, such as periodontitis and CVD, lead to an increase in systemic hs-CRP, which in turn serves as a stimulus to release MMP-9 locally (salivary) and systemically (serum) in order to protect tissues from tissue and endothelial damage caused by the inflammatory state and oxidative stress [37,38,39]. In this regard, according to the present study, Wu et al. [40] found that patients with periodontitis had significantly high serum levels of CRP and MMP-9 compared to healthy patients, especially when periodontitis was in an active phase. Moreover, modulations in host inflammatory and immune responses to periodontal infection have long been suggested to cause the increased incidence, severity, and progression of periodontitis in individuals with another systemic disorder, diabetes mellitus [41]. In an attempt to explain the exacerbated destruction of periodontal tissue in these risk groups, MMP levels have been shown to be a specific risk marker in individuals with diabetes-associated periodontitis as an anaerobic environment within the periodontal sulcus results and a gingival inflammation with a key role played by MMP-9 [42]. During systemic inflammation of systemic diseases such as CVD or diabetes, MMP-9 was mainly synthesized by neutrophils, with increased levels during inflammatory processes, leading to tissue damage to collagen-rich tissue when overproduced [43].

However, although at present there is a certain amount of preliminary evidence that analyzes serum or salivary levels such as in patients with periodontitis or with CVD, to the authors’ knowledge, there is no targeted evidence that has correlated serum and salivary levels of MMP-9 in patients with periodontitis and CVD in order to better understand the role of this mediator as an early marker of CVD or endothelial risk.

However, the present study did not show statistically significant correlations between salivary and serum levels of MMP-9. This may be due to the way the patients were enrolled, as well as the type of salivary collection techniques used in the present study. In particular, previous work has demonstrated that dental cotton rolls or unstimulated saliva can affect some assays to varying degrees depending on the type of cotton or method used [44,45,46].

In this regard, there are studies in the literature that link, even bilaterally, endothelial damage due to NO release and MMP-9. Evidence has shown that oral (salivary) MMP-9 can exert a systemic effect at the endothelial level, mediated specifically by the release of NO [33]. In this regard, periodontitis has been closely related to high levels of NO [46], so it is possible to hypothesize a close correlation between NO, MMP-9, and periodontitis, which, when associated with CVD, maybe a significant risk factor to the development of negative CVD outcomes [47]. During periodontitis, NO upregulation is linked to the host immune response present following infection by periodontal pathogenic biofilm bacteria at the level of periodontal tissues, which is strongly exacerbated during periodontitis [47,48]. However, at present, there is no full consensus regarding the effects of NO and direct oxidative stress on periodontal tissues in the presence of periodontitis. Indeed, some studies have shown high NO and MMPs in patients with periodontitis during the active phase of the disease [40,49,50,51]; otherwise, other evidence has shown low levels of NO and some classes of MMPs during periodontitis [52].

This discrepancy among the results currently present in the literature may be due to the different heterogeneity of the populations analyzed in the various studies, as well as by the number of subjects analyzed and by the possible recruitment of smokers [47]. In fact, it has been shown that cigarette smoking is closely linked to an increase in salivary NO levels [53].

Another explanation for the discrepancy between the results of the various studies may be due (as in the present study) to the sampling method and, above all, to the method of collecting and analyzing six serum and salivary levels of MMP-9. Furthermore, the reason for the different expression between serum and salivary MMP-9 may be due to the different concentration of NO between the oral and systemic environment, in which the impact of periodontitis can have different effects.

Furthermore, a possible explanation for this study’s results comes from other evidence that has shown that the immune response mediated by MMP-9 is associated with the possible presence of some heat shock proteins released during periodontitis that exert specific action at the level of T lymphocytes [50,54]. In this regard, recent studies validated MMP-9 as an essential modulator of the host’s defense mechanisms during the initial immune phase [52,53,54,55], which in turn influences, in a cascade, the entire host defense mechanism, endothelial homeostasis, and finally, CVD and periodontitis risk [56,57,58]. Furthermore, some studies demonstrated that MMPs can be lowered by some sub-antimicrobials, such as dose doxycycline. It has been shown that tetracycline can reduce MMP-8 and -9 activity in GCF and in gingival tissue, even in a much lower dosage than a traditional antimicrobial dosage used in conventional therapy [59].

However, the present study has some limitations that need to be highlighted, including the number of patients enrolled and the type of study, cross-sectional, which does not fully assess the impact of high serum and salivary levels of MMP-9, especially in the long term, with a cause-and-effect relationship. However, the statistical design used in the present study, associated with the different confounders used, allowed us to better detail the results obtained to validate the association analysis of the serum and salivary levels of MMP-9.

In the periodontal field, more and more approaches have recently been developed to evaluate the impact of mediators during periodontitis and various systemic diseases, including CVD. At present, the analysis of the salivary levels of different risk mediators opens research in the periodontal field to different broad horizons in the scientific field for the analysis and early staging of various pathologies, both oral and systemic. The results of the present study show that patients with CVD and periodontitis + CVD had elevated levels of serum and salivary MMP-9. Furthermore, the results show that the combined presence of periodontitis and CVD resulted in a synergistic effect on potentially hs-CRP-mediated MMP-9 levels compared to subjects with periodontitis and healthy subjects. The results of the present study appear highly encouraging but need to be better validated by studies on a larger number of patients and with longitudinal design in order to better understand the upregulation of MMP-9 in patients with periodontitis and CVD.

## 4. Materials and Methods

For this study, healthy subjects and patients with periodontitis and CVD were enrolled. In order to obtain a homogeneous sample, patients in a specific age group (35–65 years) and with an equal representation of both sexes were enrolled in order to reduce possible bias related to gender or different age.

The study was performed in accordance with the 2016 Helsinki Declaration on medical research. Before starting the data analysis, approval by the local International Review Board and informed consent was obtained from all participants. The study was also registered on clinicaltrials.gov (NCT03152181). The study was performed in accordance with the Strengthening the Reporting of Observational Studies in Epidemiology (STROBE) guidelines to strengthen the reporting of observational studies (Table S1).

For patients with periodontitis [60], the following inclusion criteria were adopted: (1) presence of at least 16 total teeth, (2) having at least 40% of sites with a clinical attachment level (CAL) ≥ 2 mm and a probing depth (PD) ≥ 4 mm, (3) having at least 40% of sites with bleeding on probing (BOP), and (4) having at least one periodontal site with ≥2 mm crestal alveolar bone loss confirmed on Rinn’s periapical radiograph [61]. 

Healthy patients enrolled presented no systemic disease, less than 10% of periodontal sites with BOP, no sites with PD or CAL ≥ 4 mm, and no radiographic signs of bone loss.

For patients enrolled with CVD, the inclusion criteria adopted were to be at least 18 years old, and to have a diagnosis of CVD with ≥50% stenosis of at least one coronary artery as verified by coronary angiography, or any past or ongoing percutaneous coronary intervention [62].

All the parameters were recorded in the medical record in the first visit in which the therapy for CVD or previous diagnostic tests (e.g., electrocardiogram, echocardiogram, etc.) was also evaluated. To enroll patients with periodontitis + CVD, the same criteria as the previous study groups (periodontitis and CVD) were used.

For all study subjects, the exclusion criteria were (1) use of contraceptive drugs; (2) use of antibiotics, anti-inflammatory drugs, or immunosuppressants in the last 3 months before the study; (3) alcohol consumption; (4) any severe allergies; (5) pregnancy or breastfeeding; (6) taking drugs that could cause side effects such as gingival hypertrophy or hyperplasia; and (7) any periodontal treatments in the 3 months prior to enrollment.

**Figure 3:**
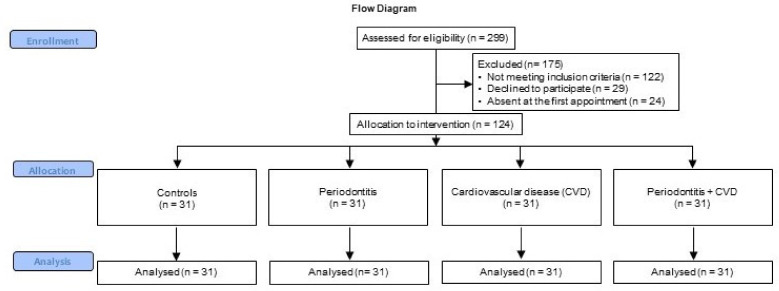
Study flowchart.

Therefore, 175 patients initially enrolled were excluded because they did not meet the inclusion criteria (n = 122), refused to participate in the study (n = 29), or were absent at the time of the first visit (n = 24). Therefore, 31 healthy subjects, 31 patients with periodontitis, 31 patients with CVD, and 31 patients with a combination of periodontitis and CVD were finally enrolled (Figure 3).

Demographic parameters (sex, age, education), body mass index (BMI), drug intake, and the presence of systemic comorbidities were collected in each patient enrolled. The presence of diabetes was assessed by the patient’s medical history or by fasting glucose ≥ 125 mg/dL. BMI was recorded by calculating the patient’s weight divided by the square of their height in kg/m^2^. All patients were also classified based on their smoking history as normal smokers, former smokers (subjects who had not smoked for ≥5 years), and non-smokers.

Periodontal charting was performed in each patient, including evaluation of clinical attachment loss (CAL), probing depth (PD), bleeding on probing (BOP), and plaque index (PI) [63]. The CAL was recorded by evaluating, using the cement-enamel junction as a reference, with PD + gingival recession. All periodontal indices were recorded in 6 sites in each tooth present in the arch.

All parameters were recorded by two independent calibrated examiners (principal and control examiner) using a periodontal probe (UNC-15, Hu-Friedy, Chicago, IL, USA).

The inter- and intra-examiner reliability for PD and CAL were assessed by intraclass correlation coefficient (ICC) analysis. The inter-examiner reliability obtained predicted an agreement for PD (ICC = 0.819) and CAL (ICC = 0.832). The intra-examiner reliability predicted agreement, for the first examiner, for PD (ICC = 0.823) and CAL (ICC = 0.814); for the second examiner, intra-examiner reliability was good for both PD (ICC = 0.839) and CAL (ICC = 0.822). Therefore, there was a good degree of reliability.

Prior to the study, a sample strength analysis was also performed to assess the correct number of patients to be enrolled for the study. The sample size was determined by considering 4 patient groups, with an effect size of 0.30 for MMP-9 (chosen primary outcome), a bilateral significance level of 0.05, a standard deviation of 1.5 [64], and a power level of 80%. It was determined that at least 29 patients per group would be needed, with a total number of 116 patients needed to reach an 80% potency level. However, calculating potential dropouts, a total of 124 patients were finally enrolled so that the power of the study was at least 80%. The power calculation was performed with statistical software (G * Power version 3.1.9.4, Universitat Dusseldorf, Düsseldorf, Germany).

### 4.1. Serological Analysis and MMP-9 Evaluation

The serum and saliva samples useful for the analysis of the study were taken from all patients between 8:00 and 10:00 in the morning. The samples were taken before the periodontal examination. All enrolled subjects were asked to refrain from drinking, eating, chewing, brushing their teeth, and other oral hygiene maneuvers in the 12 h prior to specimen collection.

For serum analysis, a venous blood sample was taken, which was immediately cooled with ice and centrifuged at 4 °C. For the collection of saliva samples, specific kits were used that included loosely chewed cotton rolls for 2 min from patients (Salivette kit, Sarsted, Verona, Italy). Saliva samples were stored immediately and then centrifuged at 4 °C. Both saliva and serum samples were stored at −20 °C.

For the analysis of MMP-9 regular immunoassays were used (R&D Systems, Minneapolis, MN, USA; Sigma-Aldrich, St Louis, MO, USA) following the manufacturer’s instructions, and hs-CRP levels were obtained by a kit of nephelometric analyses. For the remaining serum parameters (glucose, lipids, etc.), routine methods were applied.

### 4.2. Statistical Analysis

As confirmed by the Kolmogorov–Smirnov test, except for age, BMI, and salivary MMP-9, almost all the variables analyzed (e.g., fasting glucose, triglycerides, periodontal indices) did not show normal distribution. For these reasons, a non-parametric test approach was chosen for data analysis [65]. Specifically, the numerical variables were analyzed using the Kruskal–Wallis test. The Mann–Whitney test was used for the pairwise comparisons. The Bonferroni correction was applied for numerous evaluations using an α level of 0.050 divided by potential inter-group comparisons (n = 6), adopting an adjusted significance level of 0.008.

To evaluate the serum and salivary levels of MMP-9 and any statistically significant increase between groups the *p*-trend analysis was performed using the Jonckheere–Terpstra test, and the correlation analysis was performed using the Spearman correlation test, used to evaluate significant differences between hs-CRP and serum and salivary levels of MMP-9.

A univariate and multivariate linear regression analysis model was also applied to evaluate, in all patients analyzed, the possible dependence between serum and salivary levels of MMP-9 on variables such as sex, education, age, education, triglycerides, total cholesterol, BMI, CRP, and CVD medications (yes/no). In the final multivariate model, sex, age, and education were incorporated as possible confounders in order to assess whether CVD, periodontitis, or hs-CRP in serum affected MMP-9. For the evaluation of MMP-9 in saliva, the same statistical model was employed but using the salivary levels of MMP-9 as the main result. All statistical analyses were performed using statistical software (SPSS 22.0 package for Windows, SPS srl, Bologna, Italy) by setting a significant value of *p* < 0.05.

## Figures and Tables

**Table 1:** Demographic characteristics of the patients. Data are shown as a median (25th; 75th percentiles) or a number with a percentage. CVD, cardiovascular disease. * *p* < 0.001 and ** *p* < 0.001 differences vs. controls. # *p* < 0.008 differences vs. patients with CVD. §§ *p* < 0.001 differences vs. periodontitis patients. hs-CRP, high sensitivity c-reactive protein.

	Controls	Periodontitis	CVD	Periodontitis + CVD
**Age (years)**	52 (47; 57)	53 (48; 58)	52 (47; 57)	53 (46; 57)
**Gender (male/female)**	16/15	15/16	14/17	15/16
***Education level***				
**Primary school, n (%)**	12 (35.4)	13 (41.9)	15 (48.4)	14 (45.2)
**High school, n (%)**	13 (45.2)	12 (38.7)	11 (35.5)	12 (38.7)
**College/university, n (%)**	6 (19.4)	6 (19.4)	5 (16.1)	5 (16.1)
**Body mass index (kg/m^2^)**	25.4 (21.8; 27.8)	24.9 (22.4; 26.4)	25.8 (21.6; 27.1)	26.5 (22.5; 26.1)
**Fasting glucose (mg/dl)**	88.9 (82.1; 93.2)	89.7 (82.3; 105.3)	87.1 (80.6; 114.6)	90.1 (85.1; 109.2)
***Smoking***				
**Never smokers, n (%)**	25 (80.7)	27 (87.1)	28 (90.4)	26 (83.9)
**Past smokers, n (%)**	4 (12.9)	1 (3.2)	1 (3.2)	3 (9.7)
**Current smokers, n (%)**	2 (6.4)	3 (9.7)	2 (6.4)	2 (6.4)
**Current smokers—number cigarettes per day, n**	5 (3; 5)	6 (4; 7)	6 (3; 6)	5 (4; 6)
***Comorbidities***				
**Diabetes, n (%)**	-	3 (9.6) **	2 (6.4) **	2 (9.6) **
***Previous CVD***				
**Atrial fibrillation, n (%)**	-	-	6 (19.3) **,§§	5 (16.1) **,§§
**Angina pectoris, n (%)**	-	-	12 (38.7) **,§§	13 (41.9) **,§§
**Stroke, n (%)**	-	-	5 (16.1) **, §§	7 (22.6) **,§§
**Heart failure, n (%)**	-	-	6 (19.3) **,§§	5 (16.1) **,§§
***Medications***				
**Antihypertensive, n (%)**	-	-	10 (32.2) **,§§	10 (32.2) **,§§
**Statins, n (%)**	-	-	10 (32.2) **,§§	9 (29) **,§§
**Low-dose aspirin, n (%)**	-	-	7 (22.6) **,§§	7 (22.6) **,§§
**Beta blockers, n (%)**	-	-	7 (22.6) **,§§	8 (25.8) **,§§
**hs-CRP (mg/L)**	2.5 (2.1; 2.9)	3.1 (2.5; 3.9) *	5.6 (4.8; 6.2) **	6.7 (5.8; 7.1) **,§§, #
**Total cholesterol (mg/dl)**	166 (139; 181)	165 (133; 181)	172 (139; 197)	175 (178; 201)
**Triglycerids (mg/dl)**	121 (91; 145)	102 (66; 128)	141 (122; 166)	139 (103; 158)

**Table 2:** Median (25th; 75th percentile) values of the periodontal characteristics of the analyzed patients. CAL, clinical attachment level; PD, probing pocket depth; BOP, bleeding on probing; PI, plaque index. ** *p* < 0.001 differences vs. healthy patients. ## *p* < 0.001 differences vs. CVD patients. §§ *p* < 0.001 differences vs. periodontitis patients.

	Controls	Periodontitis	CVD	Periodontitis + CVD
**Number of teeth**	25 (21; 26)	19 (17; 22) **	22 (20; 25) **,§§	19 (18; 21) **,##
**CAL (mm)**	1.1 (0.8; 1.4)	3.3 (3; 3.9) **	2.2 (1.9; 2.3) **,§§	3.6 (3.1; 3.9) **,##
**% CAL 4–5 mm**	-	36.6 (35.4; 43.7) **	-	39.9 (34.8; 45.1) **,##
**% CAL ≥ 6 mm**	-	19.1 (17.7; 23.1) **	-	18.1 (16.1; 23.1) **,##
**PD (mm)**	1.4 (1.4; 1.8)	4.3 (3.6; 4.8) **	1.9 (1.7; 2.5) **,§§	3.8 (3.6; 4.45 **,##
**% PD 4–5 mm**	-	41.2 (37.9; 44.6) **	-	43.3 (41.9; 49.2) **,##
**% PD ≥ 6 mm**	-	22.9 (18.6; 24.4) **	-	23.2 (21.1; 25.9) **,§§,##
**% BOP**	7.7 (5.9; 9.1)	39.9 (35.8; 47.1) **	7.9 (5.9; 9.2) **,§§	41.6 (39.9; 47.1) **,§§,##
**PI (%)**	5.9 (4.8; 8.8)	33.2 (32.4; 35.8) **	12.3 (8.9; 11.4) **,§§	35.5 (29.9; 35.7) **,##
**Rx alveolar bone loss (mm)**	0.1 (0; 0.6)	2.7 (2.1; 3.2) **	0.2 (0.1; 0.4) **,§§	3.1 (1.9; 4.4) **,##

**Table 3:** Linear regression models that analyzed serum and salivary MMP-9 concentrations.

**Serum** **MMP-9 Levels**		**Univariate**			**Multivariate**		
	**Variable**	**Beta**	**95% CI**	***p***	**Beta**	**95% CI**	***p***
	**CVD**	0.439	0.221; 0.443	<0.001	0.124	−0.356; 0.442	0.312
	**Periodontitis**	0.274	0.179; 0.331	0.028	0.236	−0.114; 0.212	0.208
	**hs-CRP**	0.225	0.111;0.145	<0.001	0.288	0.054; 0.213	<0.001
	**Age**	−0.074	−0.287; 0.056	0.078	−0.039	−0.111; 0.315	0.287
	**Female sex**	0.118	−0.78; 0.549	0.331	0.219	−0.88; 0.428	0.112
	**Education**	−0.124	−0.151; 0.144	0.281	−0.117	−0.279; 0.412	0.247
**Salivary** **MMP-9 levels**							
	**CVD**	0.379	0.133; 0.441	<0.001	0.014	0.09; 0.115	0.366
	**Periodontitis**	0.076	−0.124; 0.248	0.428	0.012	−0.155; 0.315	0.574
	**hs-CRP**	0.084	0.112; 0.366	<0.001	0.125	0.015; 0.228	<0.001
	**Age**	−0.055	−0.110; 0.036	0.347	0.055	−0.028; 0.087	0.741
	**Female sex**	0.079	−0.112; 0.211	0.311	0.07	−0.065; 0.412	0.321
	**Total Cholesterol**	−0.058	−0.151; −0.062	0.032	−0.66	−0.041; 0.314	0.029
	**Serum MMP-9**	0.124	−0.021; 0.557	0.059	−0.038	−0.369; 0.166	0.331

## Data Availability

The study was registered at clinicaltrials.gov (NCT04152187).

## Supplementary Materials

The following are available online, Table S1: Strobe checklist.

## Sample Availability

Data Samples are available from the authors upon reasonable request.
